# Oral immunization with *Lactococcus lactis* secreting attenuated recombinant staphylococcal enterotoxin B induces a protective immune response in a murine model

**DOI:** 10.1186/1475-2859-12-32

**Published:** 2013-04-05

**Authors:** Giselli Fernandes Asensi, Nathalia Ferrari Fonseca de Sales, Fabiano Ferreira Dutra, Daniel Ferreira Feijó, Marcelo Torres Bozza, Robert G Ulrich, Anderson Miyoshi, Katia de Morais, Vasco Ariston de Carvalho Azevedo, Joab Trajano Silva, Yves Le Loir, Vânia Margaret Flosi Paschoalin

**Affiliations:** 1 Universidade Federal do Rio de Janeiro, Rio de Janeiro, RJ, Brazil; 2 Laboratory of Molecular Immunology, USAMRIID, Frederick, MD, USA; 3 Universidade Federal de Minas Gerais, Belo Horizonte, MG, Brazil; 4 INRA, UMR1253 STLO, Rennes, F-35042, France; 5 Agrocampus Ouest, UMR1253 STLO, Rennes, F-35042, France

**Keywords:** Recombinant enterotoxin B (rSEB), *Staphylococcus aureus*, Oral mice immunization, *Lactococcus lactis*, Intestinal IgA antibodies, Serum IgG antibodies, Live vaccine

## Abstract

**Background:**

*Staphylococcus aureus* is unrestrictedly found in humans and in animal species that maintain thermal homeostasis. Inadequate cleaning of processing equipment or inappropriate handling can contaminate processed food and cause severe food poisoning. Staphylococcal enterotoxin B (SEB), a potent superantigenic exotoxin, is produced by 50% of clinical isolates of *S. aureus* and is associated with massive food poisoning and with the induction of toxic shock syndrome.

**Results:**

A gene sequence encoding a recombinant SEB (rSEB), devoid of superantigenic activity, was successfully cloned and expressed in a cytoplasmic or a secreted form in the food-grade lactic acid bacterium *Lactococcus lactis*. The recombinant protein detected in the cytoplasm or in the culture medium exhibited the expected molecular mass and was recognized by a SEB-polyclonal antibody. Oral immunization with the recombinant *L. lactis* strains induced a protective immune response in a murine model of *S. aureus* infection. Immunized mice survived intraperitoneal challenge with an *S. aureus* SEB-producer strain. Counts of *S. aureus* in the spleen of rSEB-immunized mice were significantly reduced. The rSEB-immunized mice showed significant titers of anti-SEB IgA and IgG in stools and serum, respectively. Both recombinant *L. lactis* strains were able to elicit cellular or systemic immune responses in mice, with no significant difference if rSEB was produced in its cytoplasmic or secreted form. However, recombinant *L. lactis* expressing the cytoplasmic rSEB increased the survival rate of the challenged mice by 43%.

**Conclusions:**

These findings show the vaccine efficacy of *L. lactis* carrying an attenuated SEB, in a murine model, following lethal *S. aureus* challenge.

## Background

*Staphylococcus aureus* is a Gram-positive opportunistic pathogen of humans and warm-blooded animals, and is part of the commensal microbiota of the skin and nares in a significant proportion of the human population. It is a leading cause of bloodstream, lower respiratory tract, and skin and soft-tissue infections. Moreover, *S. aureus* has a wide range of virulence factors, including superantigens such as staphylococcal enterotoxins (SEs). These heat-stable toxins cause a self-limiting gastrointestinal intoxication, but parenteral exposures can cause a potentially fatal toxic shock syndrome [1-3]. Staphylococcal enterotoxin type B (SEB) is a single polypeptide of approximately 27 kDa, highly resistant to proteases [4]. As a superantigen, SEB is capable of massive activation of CD4+ lymphocytes, with subsequent secretion of cytokines and systemic inflammation [5]. Because of its remarkable toxicity and stability, SEB is considered a prime threat as a biological weapon of mass destruction [6-8]. Bacterial superantigens can be inactivated by rational site-directed mutagenesis, and these genetically altered constructs can be used for vaccine purposes [9,10]. Additionally, various vaccination regimens of an attenuated SEB mutant protein containing L45R, Y89A and Y94A were effective in a primate model against aerosolized wild-type SEB, with a correlation between survival of rhesus monkeys, antibody titers, and neutralizing antibody [11].

Lactic acid bacteria (LAB) have been considered good candidates for controlled and targeted administration of heterologous proteins to the mucosal immune system [12,13]. *Lactococcus lactis* is widely used as a starter in the dairy industry and is considered a model organism for LAB. Several genetic tools for the model LAB, *Lactococcus lactis*, were developed: transformation protocols, cloning- or screening-vectors and mutagenesis [14] (for a review) and the complete genome sequence is now available for several strains [15]. *L. lactis* has been extensively used for antigen delivery [12,16-18] thanks to genetic tools allowing antigen production in different cellular compartments (intracellular, secreted, or anchored to the cell wall) [8,19]. *L. lactis* has also been used to efficiently produce, secrete, and deliver therapeutic proteins to the mucosal tissues, specifically through the intranasal, oral, or genital mucosal surfaces [12,17]. Sufficient data are now available to support the use of recombinant LAB, in particular *L. lactis*, to deliver therapeutic proteins to the mucosal tissues [8,20]. Mucosal vaccination is able to stimulate an immune response at the site of invasion, providing a first line of defense against pathogens [13,21]. In addition, oral immunization frequently evokes both local and systemic immune responses, resulting in the effective elimination of foreign invaders [13].

The objective of this study was to explore the efficacy of an oral vaccine in C57Bl/6 mice, using *L. lactis* strains to deliver a recombinant SEB protein lacking superantigenic activity [10]. This rSEB variant was obtained after mutations in a hydrophobic binding loop, polar binding pocket, and disulfide loop (L45R, Y89A, and Y94A, respectively) without affecting the antigenic characteristics of SEB [10,11]. The humoral immune response against rSEB in mice was characterized, and its protective effect was evaluated through a challenge infection using a live SEB-producer strain of *S. aureus*.

## Results

### Engineering of recombinant *L. lactis* strains producing cytoplasmic and secreted forms of rSEB

**Figure 1:**
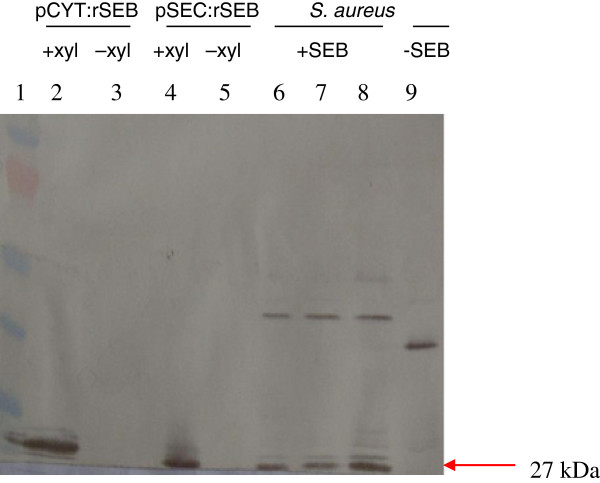
**Expression of rSEB in *Lactococcus lactis *pSEC:rSEB (secreted) and pCYT:rSEB (cytoplasm) after induction by 1% xylose, assayed in supernatants or cell pellets, respectively.** rSEB expression was analyzed by Western blot using a commercial polyclonal anti-staphylococcal enterotoxin B antibody (anti-SEB), as a probe. rSEB (27 kDa) is indicated by an arrow. Lane 1 – prestained protein molecular weight markers (Fermentas); lanes 2 and 4, -induced (+ xyl); lanes 3 and 5, non-induced (+ xyl) cultures; lanes 6, 7 and 8, cell pellets from *S. aureus* cultures + SEB (ATCC 14458 strain/SEB producer strain); lane 9, – SEB (29 strain/SEB non-producer strain). The arrow indicates the SEB of an apparent molecular mass of 27 kDa.

Since the immune response to an antigen depends on its presentation [12] we engineered *L. lactis* for the intracellular delivery of rSEB or secretion of rSEB by *L. lactis* to the intestinal mucosa. Two expression vectors were initially constructed, pCYT:rSEB and pSEC:rSEB for the cytoplasmic expression or secretion of rSEB, respectively (see Methods for details). The pCYT:rSEB vector harbors a transcriptional fusion between the ribosome-binding site (RBS*usp45*) of the *usp45* gene [22] and the DNA sequence encoding the mature moiety of rSEB, and the pSEC:rSEB harbors a transcriptional fusion between RBS*usp45* and the DNA sequence encoding the signal peptide (SP*usp45*) of Usp45 plus rSEB. In both cases, *rSEB* expression was under the control of the xylose-inducible promoter, P*xylT*[14]. The ability of *L. lactis* to secrete rSEB or to accumulate rSEB intracellularly was examined using *L. lactis* (pSEC:rSEB) and *L. lactis* (pCYT:rSEB), respectively, after induction with 1% xylose. Cell pellet and supernatant protein samples from *L. lactis* carrying pCYT:rSEB or pSEC:rSEB, respectively, were prepared from late exponential-phase cultures (cells harvested at OD_600_ 1.5), resolved by SDS-PAGE and transferred onto a PVDF membrane. Production of rSEB, in the culture medium or in the whole-cell extracts, was analyzed by Western blotting, using a commercial polyclonal anti-staphylococcal enterotoxin B antibody (anti-SEB) as a probe. A single band with the expected size for rSEB (27 kDa) – available in the Protein families database Pfam [http://www.sanger.ac.uk/Software/Pfam/search.shtml] - was identified in the cell pellet (Figure 1, lane 2) and in culture supernatants (Figure 1, lane 4) of induced *L. lactis* (pCYT:rSEB) and *L. lactis* (pSEC:rSEB), respectively. The 27 kDa polypeptide band was not detected in the samples prepared from non-induced recombinant *L. lactis* (Figure 1, lanes 3 and 5). Protein extracts of *S. aureus* ATCC 14458 (an SEB producer) and of a non-SEB producer *S. aureus* (strain from our laboratory collection) were used as positive (Figure 1, lanes 6, 7 and 8) and negative (Figure 1, lane 9) controls, respectively. Taken together, the results showed that *L. lactis* carrying (pCYT:rSEB) or (pSEC:rSEB) was able to produce rSEB intracellularly and also to secrete rSEB, both of which were properly recognized by the anti-SEB antibody.

### Protective effect of oral immunization with rSEB

**Figure 2:**
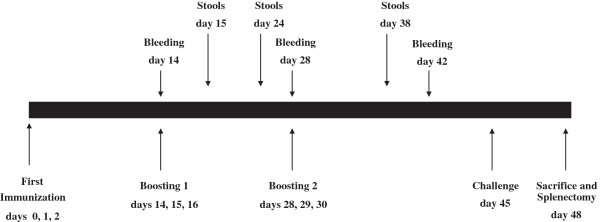
**Immunization, serum and stool collection, and challenge schedule.** To evaluate the rSEB-associated immune response in a murine model, four groups of 10 C57Bl/6 mice were tested, designated PBS, *Lactococcus lactis*, pCYT:rSEB, and pSEC:rSEB administered as shown in the schedule. Each group was immunized orally on days 0/1/2, and boosted on days 14/15/16 and on days 28/29/30. During the experiment, mice were bled three times, on days 14, 28 and 42. Stools were collected on days 15, 24 and 38. Animals were challenged by 7 × 10^8^ CFU *Staphylococcus aureus* ATCC 14458. The animals were killed at the end of the experiment on day 48, before splenectomy.

**Figure 3:**
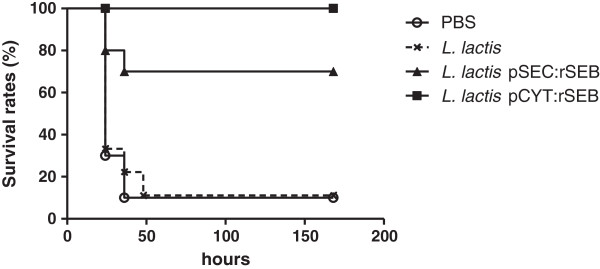
**Survival rates of immunized mice.** 10 animals in each group previously immunized with *Lactococcus lactis* pCYT:rSEB, *L. lactis* pSEC:rSEB, *L. lactis* NCDO2118, or PBS were challenged with 7 × 10^8^ CFU of *Staphylococcus aureus* ATCC 14458 (SEB producer) 14 days after the last boost.

**Figure 4:**
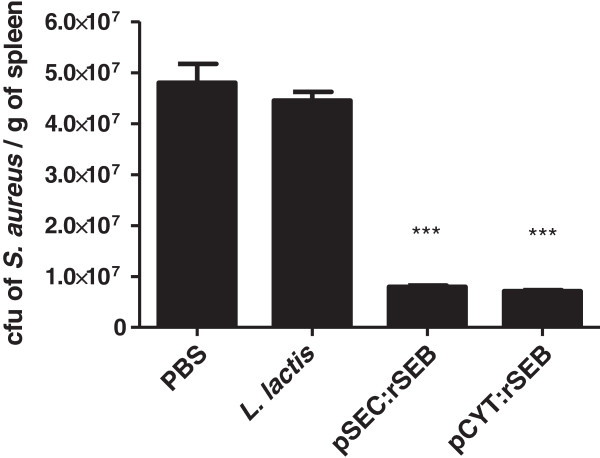
**Enumeration of *Staphylococcus aureus *in the spleen of immunized mice.** 10 animals in each group were immunized with *Lactococcus lactis* pCYT:rSEB, *L. lactis* pSEC:rSEB, *L. lactis* NCDO2118, or PBS and then challenged with 7 × 10^8^ CFU of *S. aureus* ATCC 14458 (SEB producer) on the 14th day after the last boost, as described in the Methods section. Significant results are marked with asterisks: *** p < 0.05.

To determine the efficacy of the recombinant *L. lactis* strains as a live vaccine against *S. aureus*, mice were orally immunized with 10^9^ CFU of each strain expressing rSEB, either in the cytoplasm or secreted in the intestinal mucosa. The rSEB expression was induced in each strain prior to oral administration, as described in the Methods section. Control groups were immunized with wild-type *L. lactis* NCDO2118 or with PBS. Mice received a booster immunization for three consecutive days. Fourteen days after the last immunization, the mice were challenged with a lethal dose of *S. aureus* ATCC 14458, a SEB producer strain (7 × 10^8^ CFU per mouse), by intraperitoneal (i.p.) administration (Figure 2). Fourteen days after the *S. aureus* challenge, 100% of the mice vaccinated with *L. lactis* pCYT:rSEB and 70% of those vaccinated with *L. lactis* pSEC:rSEB survived. In contrast, only 10% of the mice injected with PBS or *L. lactis* NCDO2118 (wt strain) survived the lethal challenge (Figure 3). Three days after inoculation, the number of bacterial cells in the spleens was determined. The rSEB-immunized mice presented significantly fewer *S. aureus* cells in the spleens compared to the control mice (Figure 4). Bacterial counts were not significantly different between the animals that were immunized with the cytoplasmic or the secreted form of rSEB (p < 0.01). There was no significant difference in the counts of *S. aureus* in the spleen of control mice that received PBS or *L. lactis* NCDO 2118. These results indicate that each animal immunized with *L. lactis* pCYT:rSEB and *L. lactis* pSEC:rSEB developed protection against systemic *S. aureus* ATCC 14458 infection.

In sum, oral immunization with rSEB delivered by *L. lactis* appeared to provide efficient protection and promoted survival against infection by a lethal *S. aureus* SEB producer.

### Production of anti-SEB antibody in orally immunized mice

**Figure 5:**
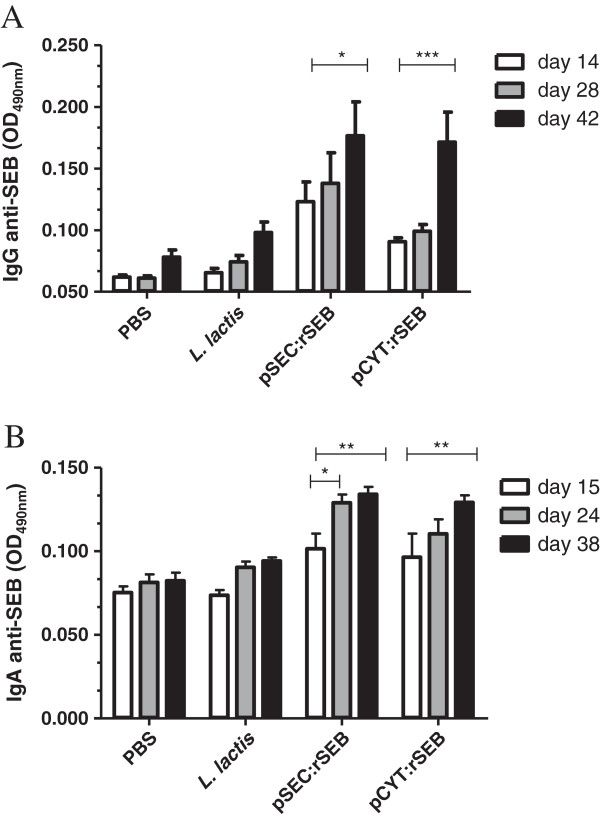
**Specific SEB antibodies.** Sera (**A**) and stools (**B**) from the different groups of mice were collected and analyzed by ELISA after the first oral administration. Immune response was assayed in mice orally immunized with recombinant *Lactococcus lactis* pSEC:rSEB or pCYT:rSEB. Significant results are marked with asterisks: *, p < 0.05; ** p < 0.01. Values were recorded as standard deviation of samples from 10 mice per group.

The production of SEB-specific IgG in serum and IgA antibody in feces was determined by specific ELISA during the time course of the immunization (Figure 5). Each animal responded with a strong, stable antibody response to SEB after the initial immunization (data not shown). The IgG antibody titer showed a significant increase 42 days after the first immunization (Figure 5A). Interestingly, immunization with *L. lactis* pSEC:rSEB induced an anti-SEB antibody production, which was stronger than that induced by *L. lactis* pCYT:rSEB, at days 14 and 28. However, specific anti-SEB antibody titers reached the same levels at day 42, in both immunizations. Fecal SEB-IgA antibodies increased significantly after immunization with rSEB-producing *L. lactis* strains with either the cytoplasmic or secreted form (Figure 5B). It appears that the intracellular presentation of rSEB by *L. lactis* or the presentation in a secreted form to the mucosal site results in a similar stimulation of local IgA antibodies.

The data presented here demonstrated that the oral administration of recombinant *L. lactis* strains carrying pSEC:rSEB or pCYT:rSEB stimulated both the serum-SEB IgG and mucosal IgA specific antibodies in mice.

## Discussion

Production of several staphylococcal proteins in *L. lactis* has been reported. However, these studies were dedicated to the development of expression-secretion systems, e.g., staphylococcal nuclease used as a reporter protein [23]; to the characterization of staphylococcal virulence factors (e.g., ClfA and FnbA [24], ClfB [25], IsdA [26]); or to increase adhesion properties of recombinant *L. lactis* strains [27]. In addition, several staphylococcal antigen candidates have been tested alone or in combination for immunization in animal models [28,29]. Proteins such as ClfA [30], recombinant forms of SEB [2] or SEC [31], as well as non-protein molecules such as capsular polysaccharides [29,32,33] are the most widely investigated *S. aureus* antigens. To our knowledge, this study reports, for the first time, the production of a staphylococcal antigen in a recombinant LAB strain to be used for oral vaccination. The two recombinant *L. lactis* strains constructed here allow for the production of a non-superantigenic rSEB, either intracellularly or secreted in the intestinal mucosa of mice, which stimulates the production of specific anti-SEB IgG antibodies in the serum and specific IgA in feces [12,34]. There is an apparent discrepancy between survival and anti-SEB IgG production when compared the CYT:rSEB and SEC:rSEB groups. It should be considered that the antigen presentation pathway (secreted versus intracellular) may also impact the quality of neutralizing antibodies, the reason that these alternative forms were tested. The immune response can reflect the production of different classes of IgG or the production of IgGs against rSEB epitopes with distinct neutralizing abilities Oral immunization with the recombinant strains induced a protective immune response against a lethal challenge with *S. aureus* ATCC 14458, an SEB producer strain, in a murine model. Interestingly, analysis of the *S. aureus* ATCC 14458 exoproteome revealed that this strain produces and secretes, in addition to SEB, other enterotoxin-like proteins such as SElK and SElQ, together with other toxins such as alpha-hemolysin and gamma-hemolysin [35]. Thus, it seems that oral immunization with a recombinant *L. lactis* producing rSEB confers a protective immunity against a strain of *Staphylococcus* that produces a variety of enterotoxins. Alternatively, one might consider that SEB, as a potent superantigen, is preponderant in the pathogenesis of SEB-producing strains. TSST1 and SEB production was indeed shown to repress exotoxin synthesis in *S. aureus*[36]. Similarly, encouraging results were obtained using a recombinant SEC mutant vaccine against mastitis. Intramuscular immunization resulted in significant protection against a challenge with live SEC-producer *S. aureus* strains in dairy cows [31]. In addition to these SE-oriented vaccines, other immunization strategies against *S. aureus* were developed, based on other staphylococcal antigens that are widely distributed among *S. aureus* strains, such as adhesins [32], IsdB [28], ClfA or exopolysaccharides [30].

Previous studies reported a protective immunization against lethal challenge with SEB after oral or nasal immunization using SEBv, a non-superantigenic variant of SEB. Significant protection was observed only when cholera toxin was used as an adjuvant [26].

The effect of the vaccination route on immunogenicity has to be considered. In a previous study, Stiles *et al.*[2] showed that immunization via the nasal route yielded higher titers of circulating IgG when compared to the oral route. An intranasal immunization route yielded a better immune response, compared to the oral route, in a live-vaccine strategy using *L. lactis* producing the HPV16 E7 antigen [12]. Whether nasal immunization with our recombinant *L. lactis* strains would confer an even better protection remains to be tested.

## Conclusions

*Lactococcus lactis,* a food-grade organism that carries and expresses an attenuated form of enterotoxin B (rSEB) appears to be an efficient delivery vehicle for immunization in mice against the toxic shock provoked by *Staphylococcus aureus*. The live vaccine can be administered orally and can be considered a promising tool to develop strategies for prevention of life-threatening toxic-shock syndrome.

## Methods

### Animals

Six- to eight-week-old C57Bl/6 mice were purchased from Cecal, Fiocruz, Rio de Janeiro, Brazil. Mice were housed in plastic cages under specific pathogen-free conditions at the Department of Immunology, UFRJ. The daily cycle consisted of 12 h light and 12 h darkness, and food and water were available at all times. The experiments were approved by the Institutional Animal Welfare Committee (approval ID: CEUA/CCS/UFRJ/IMPPG 011).

### Bacterial strains, growth conditions and plasmids

*Escherichia coli* TG1 was aerobically grown at 37°C in Luria-Bertani medium. *L. lactis* NCDO2118 was grown anaerobically at 30°C in M17 medium supplemented with 0.5% glucose. When required, antibiotics were added as follows: ampicillin (100 μg/mL) for *E. coli* and chloramphenicol (10 μg/mL) for *L. lactis*. rSEB was cloned into lactococcal plasmids pCYT:Nuc and pSEC:Nuc under the regulation of a xylose-inducible promoter, P*xylT*[17]. rSEB was produced either in the cytoplasm (CYT) or into the extracellular medium (SEC). For the sake of simplicity, these constructions are hereafter referred to as pCYT:rSEB, and pSEC:rSEB, referring to the location of the recombinant protein.

### DNA manipulations

General DNA manipulation techniques were carried out according to standard procedures [37]. DNA restriction and modification enzymes were used as recommended by the suppliers. DNA fragments were isolated from agarose gels with the Concert™ Rapid Gel Extraction System (Gibco BRL). PCR amplifications were performed using Taq DNA polymerase (Invitrogen) in a DNA thermocycler (MJ Research, Inc.). DNA plasmids from *E. coli* and *L. lactis* were isolated as previously described [37,38].

The vectors to produce cytoplasmic or secreted rSEB, pCYT:rSEB, and pSEC:rSEB, respectively, were obtained as follows: A 721-bp DNA fragment encoding rSEB was PCR-amplified from a plasmid previously described [10] and subcloned into pGEM-T (Promega) cloning vector, resulting in pGEM:rSEB. A primer pair was then designed based on rSEB sequence: 5'GGCTGCAGAGAGTCAACCAGATCCT-3' for the coding strand containing a *Pst*I restriction site (underlined) and 5'GGGAATTCTCACTTTTTCTTTGTCGT-3' for the complementary strand, containing an *EcoR*I restriction site (underlined). The rSEB fragment resulting from *Pst*I and *Eco*RI digestion of pGEM:rSEB was cloned into a *Nsi*I-*Eco*RI-cut and the purified backbone of the pCYT:Nuc expression vector, replacing the DNA sequence encoding NucB. The pSEC:rSEB vector was constructed to target the rSEB protein to the extracellular medium (i.e., secreted) of *L. lactis*, using the following procedures: the rSEB gene was PCR-amplified from pGEM:rSEB using 5'GGCTGCAGAGAGTCAACCAGATCCT-3' (including a *Pst*I site; underlined) as forward primer and 5'GGGAATTCTCACTTTTTCTTTGTCGT-3' (including an *EcoR*I site; underlined) as reverse primer. The PCR product was then *Pst*I- and *Eco*RI-digested and cloned, in frame with the signal peptide coding sequence, into the purified backbone isolated from the *Nsi*I-*EcoR*I-cut pSEC:Nuc expression vector, again replacing the DNA sequence encoding for NucB. In both cases, pCYT:rSEB and pSEC:rSEB were first obtained in *E. coli* TG1 and then transferred into *L. lactis* NCDO2118. All constructions were confirmed by DNA sequencing.

### Conditions of xylose induction

*L. lactis* strains harboring pCYT:rSEB or pSEC:rSEB were grown at 30°C overnight in 10 mL M17, containing 0.5% glucose. For xylose induction, 1 μL of the culture was transferred into 10 mL M17 medium supplemented with 1% xylose in the presence of chloramphenicol (10 ng/mL) [14].

### Protein sample preparation and Western blotting analysis

Protein samples from *L. lactis* cultures were prepared as previously described [39], except for the use of protease inhibitors and mild protein precipitation procedures. Briefly, protein extracts were prepared from 2 mL of cultures centrifuged at 17,500 × g at 4°C for 10 min. The cell pellet and supernatant were treated separately. To inhibit proteolysis in supernatant samples, 1 mM phenylmethylsulfonyl fluoride (PMSF) and 10 mM dithiothreitol (DTT) were added. Proteins were then precipitated by addition of 100 μL of 100% trichloroacetic acid. The supernatant proteins were incubated for 1 h on ice, and collected by centrifugation (17,500 × g at 4°C for 20 min). Supernatant proteins were resuspended in 50 mM NaOH, 1 mM PMSF, and 1.2 μL DTT-LB (4 mL charge buffer solution, consisting of 0.1 Tris–HCl pH 6.8, 4% SDS, 10 ml glycerol, 0.2% bromophenol blue, 50 mL distilled water q.s.p., and 1 mL of 1 M DTT). The cell pellets were resuspended in 120 μL of TES-Lys buffer (25% sucrose, 1 mM EDTA, 50 mM Tris–HCl pH 8.0 and 1 mg/mL of lysozyme), 1.2 μL DTT and 1 mM PMSF. The suspension was incubated for 30 min at 37°C, and 20% SDS, 1.2 μL DTT-LB and 1 mM PMSF were added. Supernatant or pellet proteins were separated in a 12% PAGE-SDS [40]. The gel-resolved proteins were transferred onto a PVDF membrane and probed with a commercial polyclonal anti-staphylococcal enterotoxin B antibody (anti-SEB, Sigma-Aldrich). Immunodetection was carried out using peroxidase conjugates (Sigma-Aldrich).

### Immunization and antibody detection

The animal study was conducted according to current Good Scientific Practice-principles (2000) and approved by the Ethical Committee of the UFRJ. Groups of C57Bl/6 mice (8–10 mice per group) were orally immunized with 10^9^ CFU of each recombinant strain suspended in 100 μL of sterile PBS for 3 consecutive days. The immunization protocol was repeated twice at 2-week intervals. Control groups received sterile PBS or a plasmidless *L. lactis* NCDO2118, in the same conditions. Blood samples were drawn on days 0, 14, 28 and 42 after immunization. Serum samples were examined by ELISA for IgG titers with specific antigen-binding activity after coating 1 μg/mL SEB in carbonate buffer (S4881 - Sigma-Aldrich) onto microplates. Anti-Mouse IgG Peroxidase antibody (Sigma-Aldrich) diluted 1:1000 was used as the secondary antibody. Stool samples were obtained 0, 15, 24, and 38 days after immunization and were examined by ELISA for IgA titers with specific antigen-binding activity after coating the microplate in the same way as for IgG ELISA. The secondary antibody, Anti-Mouse IgA Peroxidase antibody (A4789, Sigma-Aldrich) was diluted 1:1000.

### Inoculum for lethal challenge

*S. aureus* ATCC 14458, an SEB-producer strain, was first grown on BHI agar (Difco) for 18 h. Freshly grown colonies were suspended in BHI broth (Difco) and incubated overnight at 37°C. Bacteria were harvested by centrifugation at 4000 × g for 10 min at 4°C, and cell density was adjusted to 7 × 10^8^ CFU/mL. The inoculum size was confirmed by serial dilutions and quantitative subcultures on Baird Parker agar, and the infecting dose was established based on constructed lethality curves. The challenge dosage was established by a lethality assay, where the dosage ranged from 10^9^ CFU to 5 × 10^8^ CFU of *S. aureus* ATCC14458 administered to 6 animals with similar body mass. The concentration of 7×10^8^ CFU was able to kill approximately 70% of animals.

### Challenge tests

10 mice in each group (PBS*, L. lactis*, pSECrSEB, and pCYTrSEB) were challenged 14 days after the last booster with a lethal dose of *S. aureus* ATCC 14458 by intraperitoneal (i.p.) injection. Untreated mice (control groups) were infected in the same way. Mice were killed on the third day after challenge (day 3) and the spleen was excised, weighed, and homogenized in 1 mL sterile PBS. Homogenates were analyzed by plating 10-fold serial dilutions, in duplicate, on Baird Parker agar supplemented with egg yolk tellurite emulsion. Colonies were counted after incubation at 37°C for 18 h. Results were expressed as log of CFU/g of spleen. Mouse deaths were recorded for 15 days.

### Statistical analysis

Data were analyzed by ANOVA, followed by Bonferroni’s test to determine the significance of differences in antibody titers between the control and experimental groups. A plot of the Kaplan-Meier method was used to estimate the survival fractions, and significance was determined by a log rank test. Differences in bacterial counts in the spleen were evaluated by Student’s *t* test and Tukey’s multiple comparison test.

## Competing interests

The authors declare that they have no competing interests.

## Authors’ contributions

GFA performed all the experimental procedures and was the primary author of the manuscript; NFFS carried out the western-blotting assays; NFFFD, DDF, and MTB participated in immunization studies and challenge tests; KM, VACA, AM, and RU participated in molecular biology studies; JTS participated in the study design and data analysis; YLL participated in the coordination of the study and drafted the manuscript; VMFP conceived the study, participated in its design and coordination, and drafted the manuscript. RU critically revised the manuscript. All authors read and approved the final manuscript.

## Authors’ information

Yves Le Loir share credit for senior co-authorship of this work.
